# Site-dependent differences in the composite fibers of male pelvic plexus branches: an immunohistochemical analysis of donated elderly cadavers

**DOI:** 10.1186/s12894-018-0369-9

**Published:** 2018-05-22

**Authors:** Kuniyasu Muraoka, Shuichi Morizane, Keisuke Hieda, Masashi Honda, Takehiro Sejima, Gen Murakami, Shin-ichi Abe, Atsushi Takenaka

**Affiliations:** 1 Department of Urology, Tottori University Faculty of Medicine, Yonago, Japan; 2 Department of Urology, Hiroshima University Faculty of Medicine, Hiroshima, Japan; 3 Division of Internal Medicine, Iwamizawa Kojin-kai Hospital, Iwamizawa, Japan; 4 Department of Anatomy, Tokyo Dental College, Tokyo, Japan; 5 Division of Urology, Department of Surgery, Tottori University Faculty of Medicine, 36-1 Nishi-cho, Yonago, 683-8504 Japan

**Keywords:** Pelvic autonomic nerve plexus, Fiber composition, Neuronal nitric oxide synthase, Vasoactive intestinal polypeptide, Tyrosine hydroxylase

## Abstract

**Background:**

Although the pelvic autonomic plexus branches are considered to be a mixture of sympathetic and parasympathetic nerves, little is known regarding the composite fibers of the pelvic plexus branches. This study aimed to investigate the immunohistochemical features of sympathetic and parasympathetic nerves in the pelvic autonomic plexus branches.

**Methods:**

Using 10 donated elderly male cadavers, the detailed topohistology of nerve fibers at and around the bladder, seminal vesicle, prostate, and rectum was examined. Neuronal nitric oxide synthase (nNOS) and vasoactive intestinal polypeptide (VIP) were used as parasympathetic nerve markers; tyrosine hydroxylase (TH) was used as a sympathetic nerve marker. The myenteric plexus of the colon was utilized as a positive control.

**Results:**

Most nerve fibers in the bladder, seminal vesicle, prostate, and rectum were both nNOS- and TH-positive. Thus, pelvic plexus branches were classified into two types: 1) triple-positive mixed nerves (nNOS+, VIP+, TH+, thick myelinated fibers + or -) and 2) double-positive mixed nerves (nNOS+, VIP-, TH+, thick myelinated fibers + or -). Notably, triple-positive nerves were localized within the posterosuperior part of the plexus (near the rectum) and travelled anteroinferiorly toward the posterolateral corner of the prostate. The posteriorly and inferiorly located nerves were predominantly composed of parasympathetic, rather than sympathetic, fibers. In contrast, nerve fibers within and along the bladder and seminal vesicle contained either no or few VIP-positive nerves. These superiorly located nerves were characterized by clear sympathetic nerve dominance.

**Conclusions:**

The nerves of the pelvic plexus branches were clearly classified into nerves around the bladder and seminal vesicle (VIP-negative) and nerves around the prostate (VIP-positive). Although nNOS- and VIP-positive nerve fibers are candidate cavernous nerves, cavernous nerve identity cannot be definitively concluded for these nerves in the periprostatic region.

## Background

Autonomic innervation of the pelvic viscera is formed by sympathetic fibers from the inferior hypogastric plexus and parasympathetic fibers from the pelvic splanchnic nerves [1, 2]. The genital organs and lower urinary tract are controlled by the autonomic nervous system, as well as the somatic nerves. Because functional disruptions, such as urinary incontinence and sexual dysfunction, represent important determinants of quality of life, detailed anatomical studies of the pelvic neuroanatomy are essential to preserve continence, erection, and bladder function after pelvic surgery [3, 4].

Immunohistochemistry is widely applied in pelvic nerve research. However, as evaluation of the periprostatic nerve distribution is typically performed using surgically acquired specimens [5–7], the staining of a large area, including adjacent organs, is generally not possible. Therefore, to investigate the anatomy of the pelvic plexus, use of the entire pelvic block of cadavers is ideal. Nitric oxide synthase (nNOS) and vasoactive intestinal polypeptide (VIP) are used as parasympathetic nerve markers, whereas tyrosine hydroxylase (TH) is used as a marker of sympathetic nerves [8]. Recent reports have demonstrated that, in the human pelvic floor, a portion of the pelvic plexus branches contained VIP-, nNOS-, and TH-positive nerve fibers [9–11].

The aim of this study was to investigate the topohistology of three types of pelvic nerve fibers (nNOS-, VIP-, and TH-positive fibers) by comparison of the periprostatic nerve configuration and distribution with that of adjacent regions.

## Methods

This study examined 10 donated male cadavers with a mean age of 73 years (range, 64–82 years). The cause of death was either ischemic heart failure or intracranial bleeding; none of the cadavers had undergone abdominal or pelvic surgery, as confirmed by review of patient medical histories, as well as by macroscopic observation of the abdominopelvic cavity. The 10 cadavers were donated to Tokyo Dental College for research and education on human anatomy in accordance with their consent, and their use in research was approved by the Ethics Committee of Tottori University Faculty of Medicine. The study was performed in accordance with the provisions of the Declaration of Helsinki 1995, as revised in Edinburgh in 2000.

The donated cadavers had been fixed by arterial perfusion of 10% *v*/v formalin solution, then stored in 50% v/v ethanol solution for > 3 months. From each of the cadavers, a large tissue block, including the bladder, seminal vesicle, urethra, prostate, and rectum, as well as any connective tissue around these viscera, was prepared. After bisection along the midsagittal line, each of the hemiblocks was cut into 15-mm thick sections; then, routine procedures for paraffin-embedded histology were performed. From each of the macroslices, large horizontal or sagittal sections (70 × 50 mm) were prepared at 2–3-mm intervals, then stained with hematoxylin and eosin (H&E). After reviewing the large sections to identify target regions, sections (50 × 20 mm) for immunohistochemistry were cut, in close proximity to the former plane. Ultimately, we obtained 2–5 large sections and 8–20 standard-sized sections from a single paraffin block containing a 15-mm-thick slice.

Most sections were stained with H&E, whereas others were stained via immunohistochemistry and elastic-tissue Masson staining (a variation of Masson-Goldner staining). Primary antibodies for nerve immunohistochemistry were used based on the methods of Hinata et al. [12]; these comprised mouse monoclonal anti-human S100 protein (1:200 dilution, Dako Z0311; Dako, Glostrup, Denmark), rabbit polyclonal anti-human nNOS (1:200 dilution; Cell Signaling Technology, Beverly, MA, USA), mouse monoclonal anti-human VIP (1:100 dilution, Santa Cruz sc25347; Santa Cruz, CA, USA), and rabbit polyclonal anti-human TH (1:500 dilution, Millipore-Chemicon ab152; Temecula, CA, USA). When possible, the four immunohistochemical stains were applied on adjacent sections; occasionally, non-adjacent sections were sometimes used because of failed immunostaining. As a positive control for immunohistochemistry, the myenteric plexus of the descending colon was acquired from the 10 specimens; the colic myenteric nerves invariably exhibit a dense distribution of nNOS-/VIP-coreactive nerve fibers, even in centenarians [13]. The secondary antibody was labeled with horseradish peroxidase (HRP), and antigen-antibody reactions were detected via the HRP-catalyzed reaction with diaminobenzidine. The immunohistochemistry-labeled samples were counterstained with hematoxylin. Negative controls comprised samples without primary antibody.

## Results

**Fig. 1:**
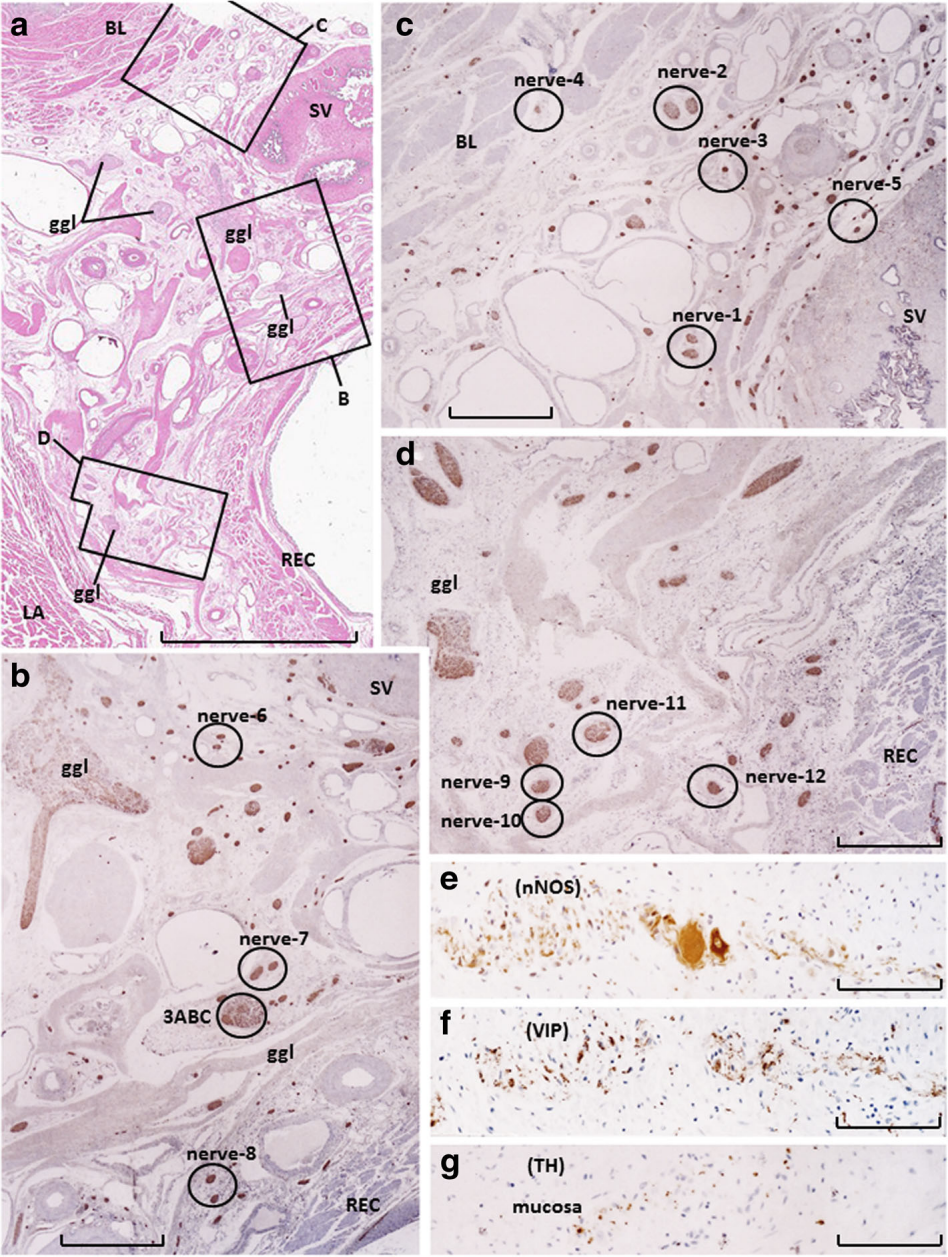
Topohistology of nerves for immunohistochemistry. Near-horizontal sections of specimens from a 76-year-old man. Panel **a** (hematoxylin and eosin staining; scale bar, 10 mm) displays topographical anatomy, including the bladder (BL), seminal vesicle (SV), rectum (REC), and levator ani muscle (LA). There are five ganglion cell clusters (ggl) in the panel. Panel **b** (an area between the seminal vesicle and rectum), panel **c** (an area between the bladder and seminal vesicle), and panel **d** (an area between the rectum and levator ani muscle), corresponding to squares **b**, **c**, or **d** within panel **a**, exhibit immunohistochemistry of S100-labeled nerves that are shown in Figs. 2, 3, 4 and 5. Scale bars in panels **b**–**d** are 1 mm. Panels **e**, **f**, and **g** (nearby sections; scale bars, 0.1 mm) depict the colic myenteric plexus in the same specimen as the positive control: the plexus strongly expresses nNOS and VIP, whereas TH-positive cells are scarce

**Fig. 2:**
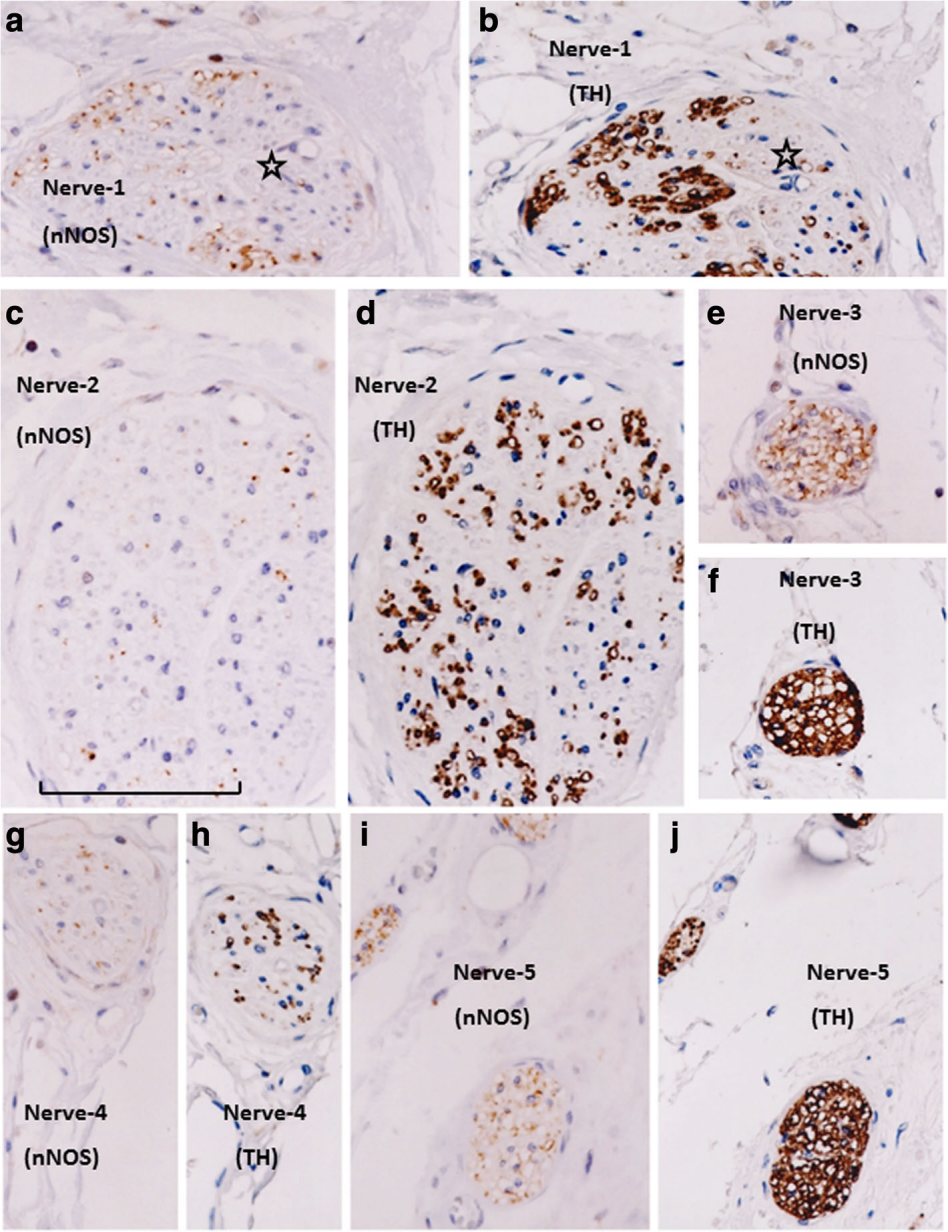
Immunohistochemistry of nerves in an area between the bladder and seminal vesicle (anterosuperior group of the pelvic plexus branches). Topographical nerve anatomy is shown in Fig. 1c. Panels **a**, **c**, **e**, **g**, and **i** exhibit nNOS immunostaining, while panels **b**, **d**, **f,** and **i** display TH immunostaining. Panels **a** and **b** (or **i** and **j**) are adjacent sections, whereas panels **c** and **d** (or **e** and **f**, **g** and **h**) are proximal sections. These nerves did not contain fibers that were reactive for VIP (not shown). In all of these nerves, nNOS- and TH-positive fibers appear to be intermingled, without clear localization. However, in panels **a** and **b**, triple-negative areas (putative myelinated sensory fiber-dominant areas; stars) are visible. Panels **g** and **h** display nerves in the bladder detrusor, while panels **i** and **j** show nerves attaching to the seminal vesicle. Nerves along the seminal vesicle are characterized by an abundance of TH-positive fibers. In contrast, bladder detrusor nerves contain an abundance of triple-negative fibers (putative sensory fibers). All panels were prepared at the same magnification (scale bar in panel **f**, 0.1 mm)

**Fig. 3:**
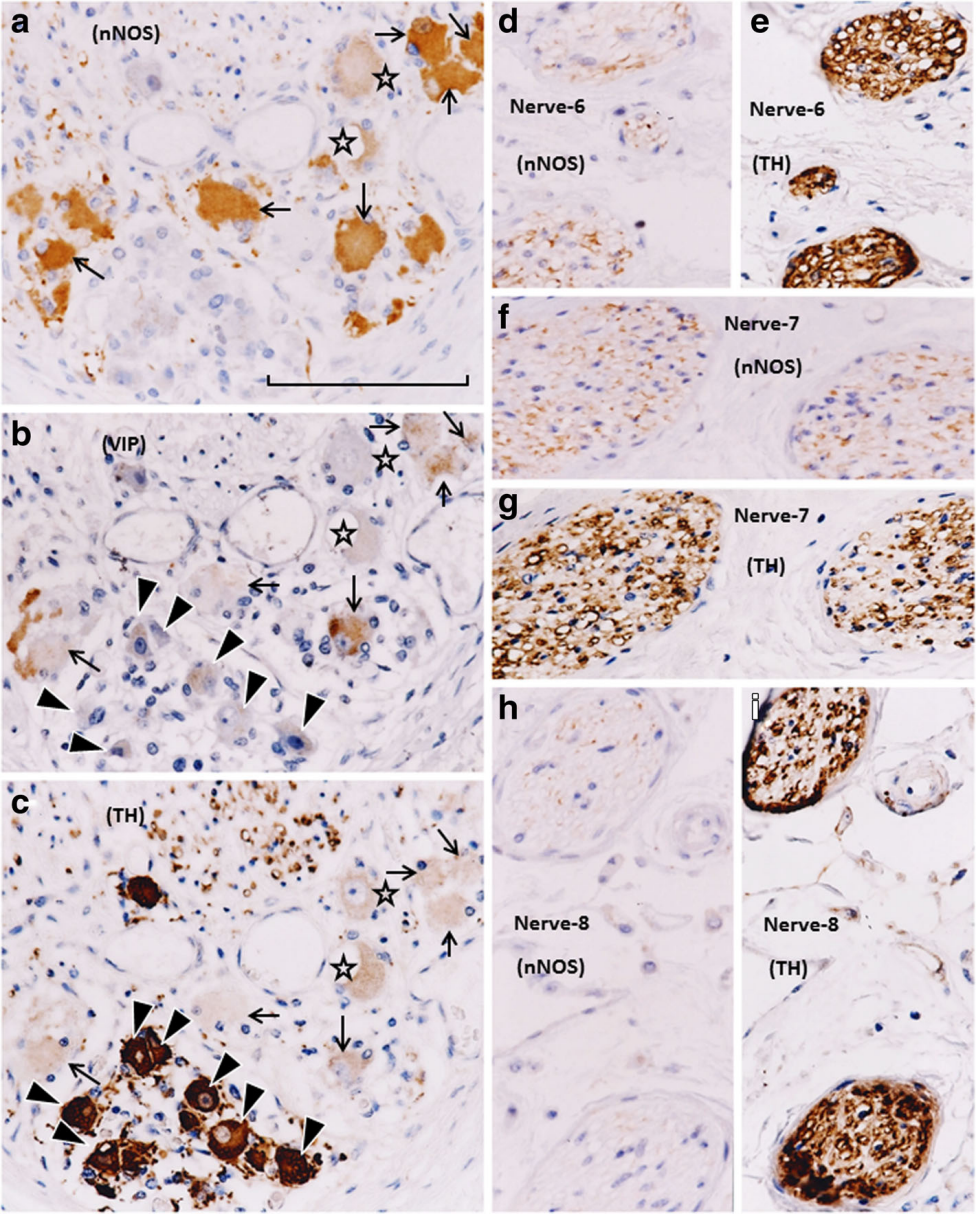
Immunohistochemistry of nerves in an area between the rectum and seminal vesicle (middle and superior group of the pelvic plexus branches). Topographical nerve anatomy is shown in Fig. 1b. Panels **a**, **d**, **f**, and **h** display nNOS immunostaining, while panels **c**, **e**, **g**, and **i** exhibit TH immunostaining. Only panel **b** displays VIP immunostaining: the other nerves in this figure did not contain VIP-positive fibers (not shown). Panels **a**–**c** (or **f** and **g**; **h** and **i**) are adjacent sections, whereas panels **d** and **e** are proximal sections. Arrows, arrowheads, and stars in panels **a**–**c** indicate ganglion cells corresponding to each panel: two ganglion cells (stars) appear to be negative for all three markers. All VIP-positive cells (panel **b**) appear to express nNOS (panel **a**). All TH-positive cells (panel **c**) do not express either nNOS or VIP. In all of these nerves, nNOS- and TH-positive fibers appear to be intermingled and do not exhibit a clear localization. All panels were prepared at the same magnification (scale bar in panel **a**, 0.1 mm)

**Fig. 4:**
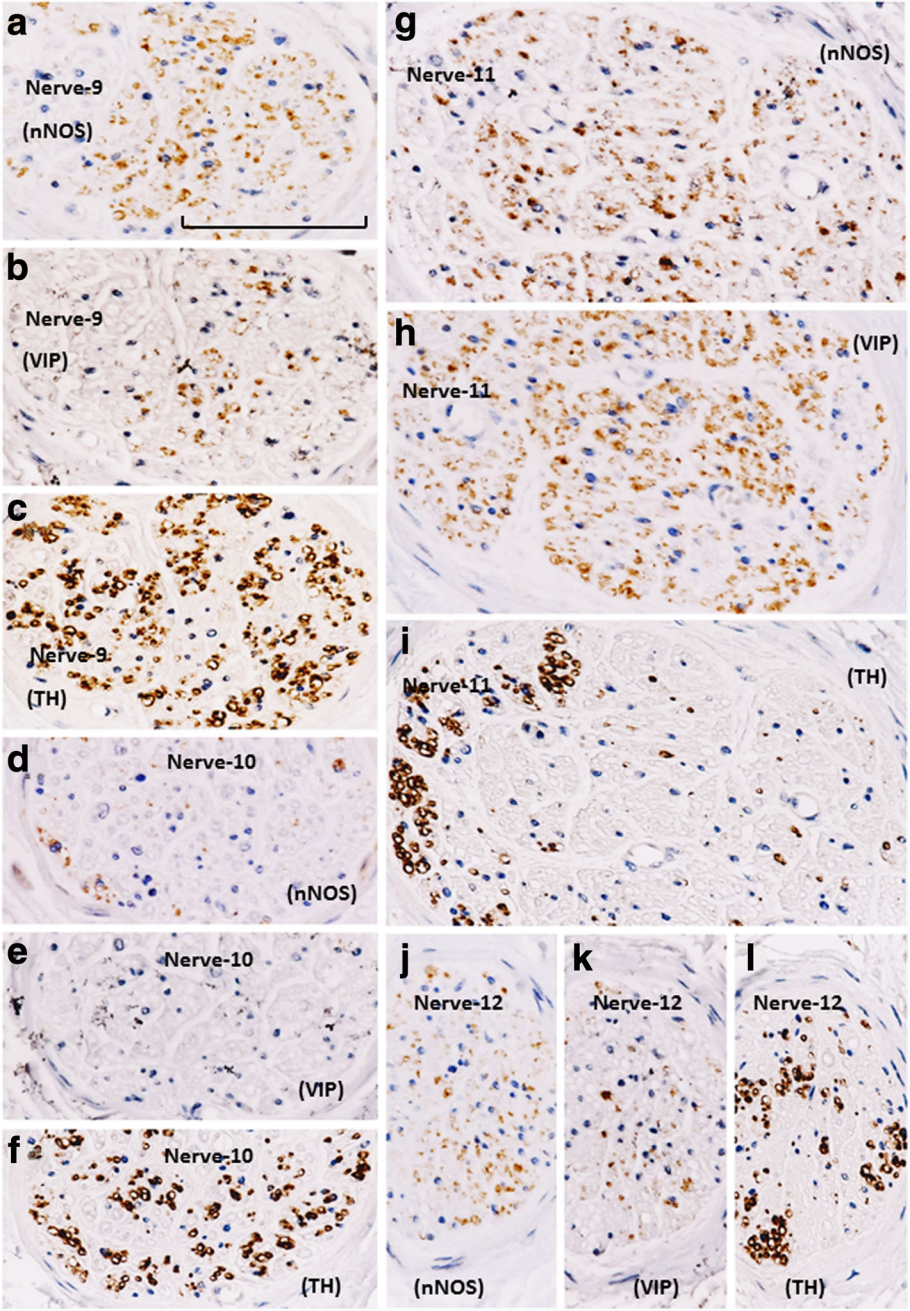
Immunohistochemistry of nerves in an area between the rectum and levator ani muscle (posterosuperior group of the pelvic plexus branches). This area is characterized by the existence of VIP-positive nerves. The topographical relationship of the four nerves is shown in Fig. 1d. Panels **a**, **d**, **g**, and **j** display nNOS immunostaining; panels **b**, **e**, **h**, and **k** exhibit VIP immunostaining; panels **c**, **f**, **i**, and **l** exhibit TH immunostaining. One of the four nerves (panel **e**) does not express VIP. In a single nerve, panel **d** does not correspond to the site shown in panels **e** and **f**, as nNOS- (or TH-) positive nerves are restricted to the upper (or lower) side of the panel. VIP-positive fibers appear to be intermingled with nNOS-positive fibers. In panel **i**, TH-positive fibers are restricted to the left-hand side of the nerve. All panels were prepared at the same magnification (scale bar in panel **a**, 0.1 mm)

**Fig. 5:**
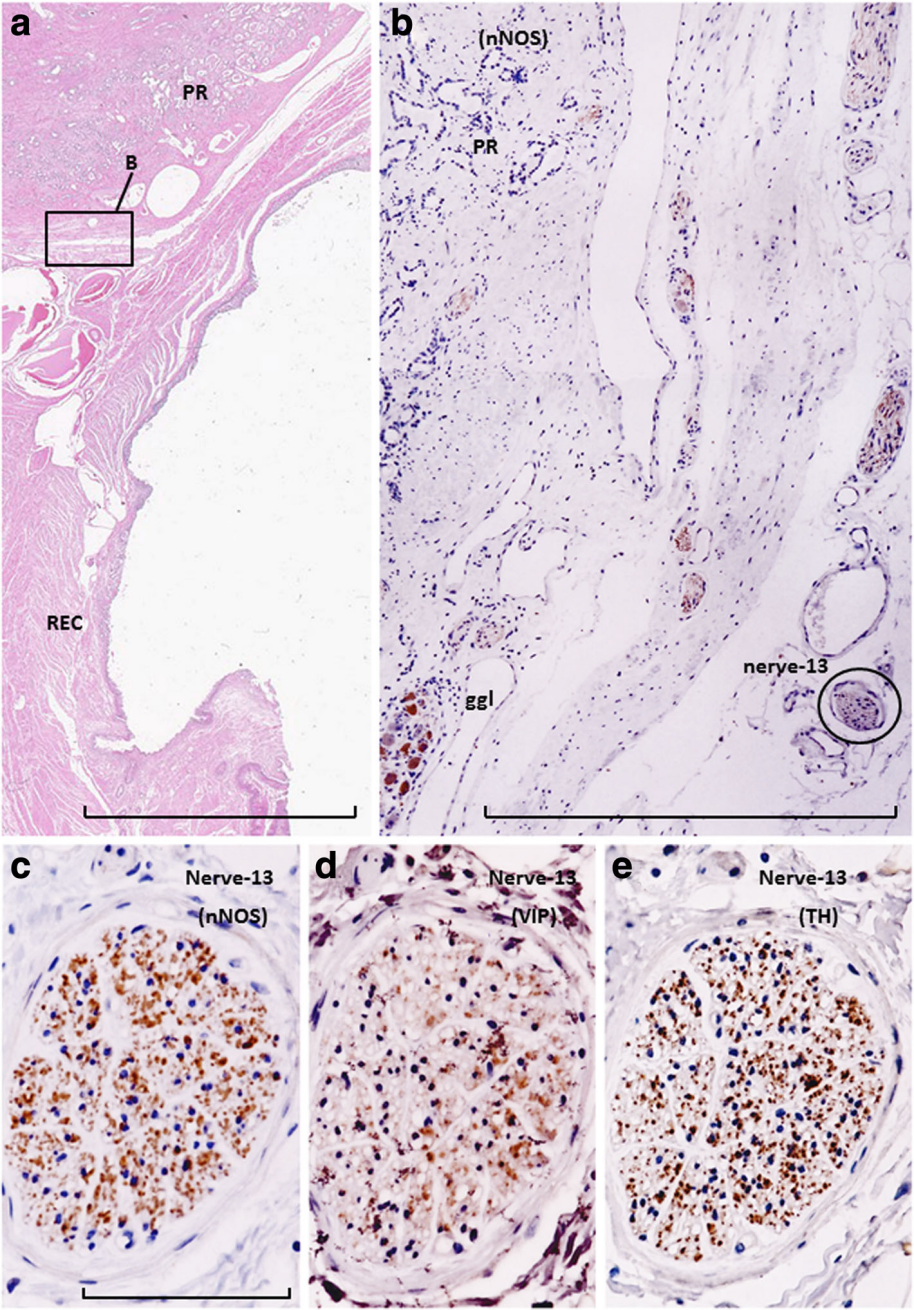
Immunohistochemistry of nerves at and along the posterolateral corner of the prostate (posteroinferior group of the pelvic plexus branches). Near-sagittal sections of specimens from a 77-year-old man. Panel **a** (HE staining; scale bar, 10 mm) displays the topographical anatomy of nerves within the prostate (PR) and rectum (REC). After rotation, a square in panel **a** is shown in panel **b**. Panel **b** displays nNOS immunostaining in a part of the periprostatic nerves: at this lower magnification (scale bar, 1 mm), strong expression of nNOS can be identified. Panels **c**, **d**, and **e** (adjacent sections), corresponding to a nerve indicated by a circle in panel **b**, show nNOS, VIP, and TH immunostaining, respectively (scale bar in panel **c**, 0.1 mm). The periprostatic nerve contains abundant nNOS- and TH-positive fibers, but VIP-positive fibers are rare

The tissue blocks that were prepared for horizontal sections contained the posterior part of the bladder, nearly the entire seminal vesicle, the superoposterior part of the prostate, the anterior wall of the rectum, and parts of the levator ani muscle. The horizontal sections were suitable for a better understanding of the topographical anatomy in and around the bladder and seminal vesicle. However, the periprostatic neurovascular bundle could not easily be included in a block because of the large mass of the prostate. Thus, to easily identify the periprostatic nerves, sagittal sections were necessary. A complete set of observations of most branches of the pelvic plexus was performed in two specimens (all horizontal sections), whereas in the other eight specimens (combination of horizontal and sagittal sections), either the superior or inferior group of nerves was examined. Figures 1, 2, 3, 4 and 5 were prepared from a combination of observations from two specimens.

In all 10 cadavers, the colic myenteric plexus expressed all three markers (nNOS, VIP, and TH; Fig. 1), although TH expression was typically weak and found in very few fibers. For descriptive purposes, the pelvic plexus branches to the urogenital organs were divided into four areas: 1) the middle and superior area between the seminal vesicle and rectum (Fig. 1b); 2) the anterosuperior area between the bladder and seminal vesicle (Fig. 1c); 3) the posterosuperior area between the rectum and levator ani muscle (Fig. 1d); and, 4) the posteroinferior area at and along the posterolateral corner of the prostate (Fig. 5b). The third group of nerves traveled anteroinferiorly toward the posterolateral corner of the prostate, such that the third area appeared to be connected to the fourth area. All four areas of nerves exhibited ganglion cell clusters, but the second and third areas (3–10 clusters per horizontal section) contained greater numbers than the others (1–5 clusters per horizontal section). Numbers of cut nerve profiles per mm^2^ in each horizontal section ranged from 15 to 25 in the first area, 22 to 38 in the second area, 8 to 13 in the third area, and 5 to 18 in the fourth area. Therefore, nerve density was consistently highest in the first (anterosuperior) area of nerves, located between the bladder and seminal vesicle.

More than 70% of nerves in the anterosuperior area were thin (< 0.05-mm diameter), and the density was higher along the seminal vesicle than the bladder (Fig. 1c). In these nerves, TH-positive sympathetic fibers were much more abundant than parasympathetic fibers, especially in the thinner nerves (Fig. 2). The proportion of TH-positive fibers in the nerve along the seminal vesicle was much higher than the corresponding proportion along the bladder. Sympathetic nerve dominance was also observed in the first (middle and superior) area between the seminal vesicle and rectum (Fig. 3). In contrast to the first and second areas, nerves in the third (posterosuperior) and fourth (posteroinferior) areas contained VIP-positive fibers, although the number per section was consistently lower than for nNOS-positive nerves (Figs. 4 and 5). Furthermore, in these posterior areas, the number of nNOS-positive fibers was often equal to or greater than the number of TH-positive fibers. In addition, with respect to intra-organ nerves, both nNOS-positive fibers and TH-positive fibers were present; these were adjacent to and alongside the glands and mucosa of the prostate, seminal vesicle, and bladder. Similarly, the smooth muscles of the prostate and seminal vesicle contained a very high proportion of TH-positive fibers, as well as multiple nNOS-positive fibers. However, immunoreactive nerves were not found in the bladder detrusor smooth muscles. Other areas of nerves were characterized according to sympathetic nerve dominance. Of note, in all four areas, the intrapelvic nerves consistently contained both nNOS-positive and TH-positive fibers. In contrast, the pelvic nerve branches, such as those surrounding the levator ani muscle, did not contain either nNOS- or VIP-positive fibers (data not shown). Ganglion cells, albeit rare, contained triple-negative fibers (Figs. 2a, b; 3a, b, c).

In a ganglion cell cluster (Figs. 3a, b, c; 5b), nNOS-positive ganglion cells were consistently highest in number, followed by TH-positive cells. Some nNOS-positive cells also appeared to express VIP when observed in serial sections. The maximum diameter of cell bodies ranged from 15 to 25 μm, irrespective of their sympathetic or parasympathetic function. TH-positive cells were found either intermingled with, or in clusters separated from, nNOS-positive cells (Fig. 3c).

**Fig. 6:**
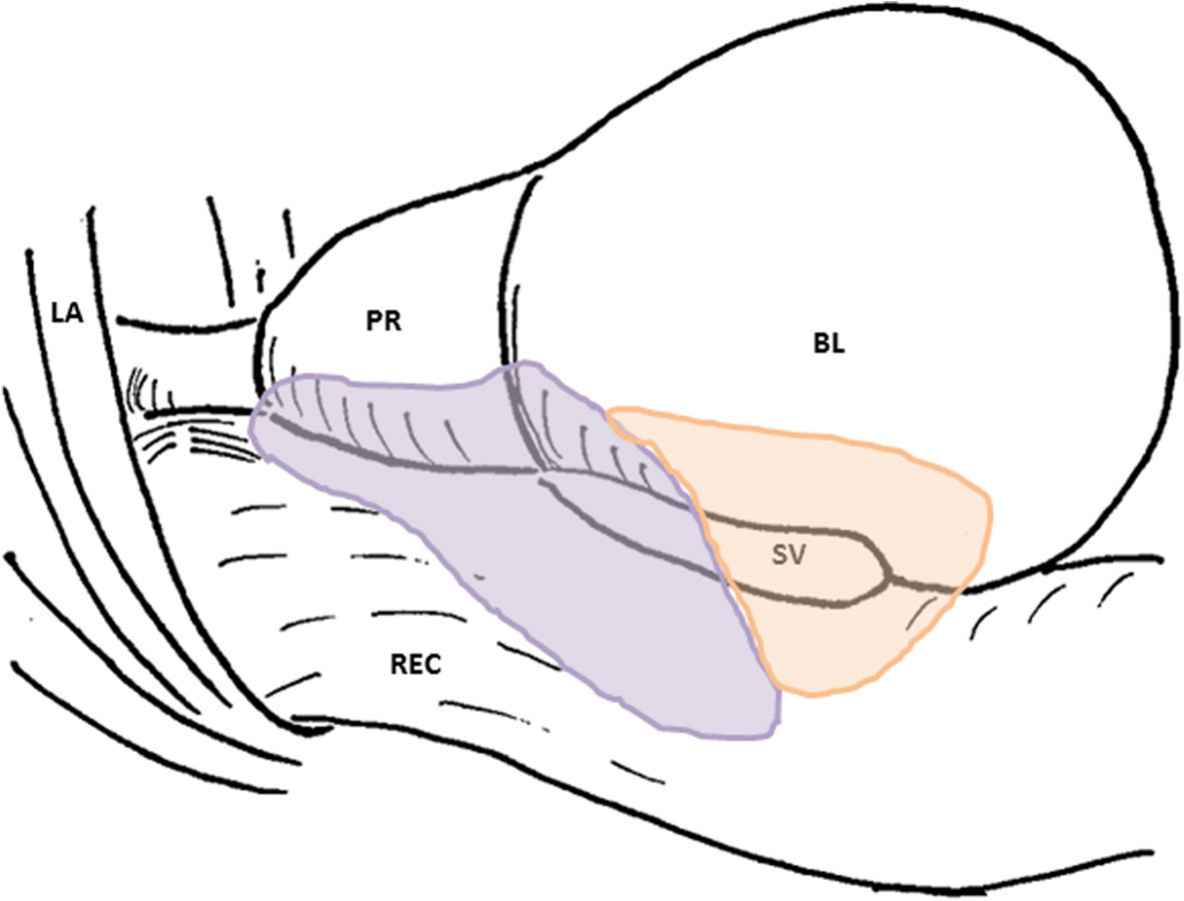
Schema of pelvic plexus branches. The dominance of VIP-positive nerve fibers varied by site; nerves of the pelvic plexus branches were clearly classified into two types. The purple and orange areas indicate triple-positive mixed nerves (nNOS+, VIP+, TH+) and double-positive mixed nerves (nNOS+, VIP-, TH+), respectively. VIP-positive nerve fibers are distributed to the posterosuperior area between the rectum and levator ani, as well as to the posteroinferior area at and along the posterolateral corner of the prostate. BL, bladder; PR, prostate; SV, seminal vesicle; LA, levator ani muscle; REC, rectum

Sympathetic nerve fibers were most frequently observed in the superiorly located branches of the pelvic plexus near the bladder and seminal vesicle; in contrast, parasympathetic fibers, a portion of which expressed VIP, were dominant in the inferiorly or posteriorly located nerves near the prostate. Based on these observations, the pelvic plexus branches were classified into two types: 1) triple-positive mixed nerves (nNOS+, VIP+, TH+, thick myelinated fibers + or -); and, 2) double-positive mixed nerves (nNOS+, VIP-, TH+, thick myelinated fibers + or -). Figure 6 depicts representative pelvic plexus branches, including both triple-positive mixed nerves and double-positive mixed nerves. The composition of nerve fibers shown in Figs. 1, 2, 3, 4 and 5 is summarized in Table 1.

**Table 1:** Summary of composite nerve fibers of the pelvic plexus branches

	nNOS	VIP	TH
The anterosuperior group between the bladder and seminal vesicle			
nerve-1	++	–	++
nerve-2	+	–	++
nerve-3	+++	–	+++
nerve-4	+	–	+
nerve-5	+++	–	+++
The middle and superior group between the seminal vesicle and rectum			
nerve-6	+++	–	+++
nerve-7	+++	–	+++
nerve-8	+++	–	+++
The posterosuperior group between the rectum and levator ani			
nerve-9	++	+	+++
nerve-10	+	–	++
nerve-11	+++	++	+
nerve-12	++	+	+
The postero-inferior group at and along the posterolateral corner of the prostate			
nerve-13	+++	+	+++
Nerves to BL	+	–	+
Nerves to SV	+	–	+++
Nerves to PR and CTs	+++	+ or ±	+

+, > 10 positive nerve fibers were seen in the nerve; ++, positive nerves occupied 30–70% of a cross-sectional area of the nerve; +++, positive fibers occupied nearly all parts of the nerve with a high density

*BL* bladder, *CTs* cavernous tissues, *PR* prostate, *SV* seminal vesicle

## Discussion

This study focused on the pelvic plexus branches, from the lateral site of the bladder, seminal vesicle, and rectum, to the posteroinferior site at and along the posterolateral corner of the prostate, in elderly male cadavers. Although limited regions of the peripheral pelvic plexus were examined, several patterns were identified in the fiber composition of the pelvic plexus branches. Nerves in the examined region consistently included TH-positive fibers; further, many of the plexus branches were both nNOS- and TH-positive. Notably, the anterosuperior area (between the bladder and seminal vesicle) and the middle and superior area (between the seminal vesicle and rectum) did not contain VIP-positive nerve fibers. On the contrary, the posterosuperior area (between the rectum and levator ani muscle) and the posteroinferior area (at and along the posterolateral corner of the prostate) contained VIP-positive nerve fibers. The hypogastric and pelvic splanchnic nerves both contain nNOS-, VIP-, and TH-positive nerves [14, 15]. Kraima et al. [15] reported that VIP-positive fibers were not present in tissues isolated from the lumbar sympathetic chain; thus, areas that do not include VIP-positive nerves may be supplied by the lumbar sympathetic chain. In addition, a non-parasympathetic pattern [−, −, +] was not evident in the plexus branches, but was observed both inside and outside of the levator ani muscle; these nerves appeared to be pudendal nerve branches. Few triple-negative fibers were observed; these are candidate pure sensory nerves [10].

TH is the rate-limiting enzyme in the synthetic pathway of norepinephrine, a neurotransmitter that is found in peripheral sympathetic nerves and their related ganglia; thus, TH is often used as a marker of sympathetic nerves [10, 14–17]. Both nNOS and VIP have been used as parasympathetic nerve markers of the pelvic plexus branches [8–11]; nNOS is found in peripheral parasympathetic nerves and catalyzes the formation of nitric oxide [18], whereas, VIP is generally considered to be the primary transmitter released from cholinergic smooth muscle vasodilator and secretomotor fibers [19].

With regard to lower urinary function, the bladder neck is innervated by dense noradrenergic nerves, which have been shown to cause smooth muscle contraction and subsequent closure of the bladder neck during the storage of urine [20]. During micturition, inhibition of the sympathetic pathway may lead to opening of the bladder neck and prostatic urethra [21]. Additionally, high NOS activity was found in the urethra, whereas intermediate activity was found in the bladder neck, and comparatively low activity was found in the detrusor muscle. VIP-containing nerves form a dense subepithelial plexus and project to the detrusor muscle bundles of the bladder [22]. VIP plays an important role in bladder neck opening by promoting relaxation of the smooth muscle [20]. With regard to sexual function, TH- and VIP-positive nerve fibers are very abundant in the human prostate [23, 24] and are considered to play a role in the expulsion of the contents of the prostate gland during seminal emission, as well as during ejaculation, which is largely under adrenergic control [21]. Sympathetic innervation mediates corporeal vasoconstriction and corporeal smooth muscle contraction; further, it causes penile detumescence after orgasm, and (in the absence of sexual arousal) maintains the penis in the flaccid state [25]. The perivascular and trabecular nerve fibers within the corpus cavernosum exhibit positive immunostaining for both nNOS and VIP [16, 26]. Nitric oxide is released by parasympathetic fibers and is a potent vasodilator associated with the physiology of the male erection. Nitric oxide and VIP participate in the erectile process via activation of the nitric oxide/cGMP and adenylyl cyclase/cAMP pathways, respectively [23, 24].

The present study demonstrated that nNOS-positive fibers are present in the pelvic plexus branches, as well as in the prostate, seminal vesicle, and bladder; further, the number of cut nerves per mm^2^ decreases with proximity to the periphery. Ganzer et al. reported that nerve planimetry, using a polyclonal antibody against the neural protein S100, revealed that 75% of nerves from the seminal vesicles do not reach the striated urethral sphincter level along the prostate [27]. Thus, the nerves around the prostatic apex may be the remaining nerves of the pelvic plexus, after distribution to the prostate, seminal vesicle, and bladder. Although nNOS immunoreactivity has been used to identify cavernous nerves, previous data, suggesting that nNOS-positive periprostatic nerves are cavernous nerves, may have been overstated.

VIP immunoreactivity is also found in the human penis, where the largest concentrations of VIP are present in the cavernosum body [28]. The present study demonstrated that approximately 10% of nerve fibers progressing toward the prostate and cavernous tissues were VIP-positive. Hinata et al. reported that there were few VIP-positive fibers adjacent to, and posterior to, the rhabdosphincter area [11]. Ehmke et al. reported that > 50% of perivascular nerve fibers and > 90% of trabecular nerve fibers within the corpus cavernosum stained positive for both nNOS and VIP. Furthermore, NOS/VIP immunoreactivity was reduced (diabetes) or absent (lesion of the cavernous nerve) in penile tissue taken from patients with neurogenic impotence [26]. Although nNOS- and VIP-positive nerve fibers from the prostatic apex toward the periphery are regarded as candidate cavernous nerves, there may be considerable interindividual variation in nNOS and VIP immunoreactivity, corresponding to erectile function.

Many excellent studies have been published regarding the macro- and microscopic anatomy of the pelvic autonomic nerve plexus and its branches. Takenaka et al. reported that the main route of the cavernous nerve branches from a location near the root of the pelvic splanchnic nerves, then joins in a spray-shaped distribution to the central area of the neurovascular bundle, travelling along the distal side of the pelvic plexus [2]. The distal pelvic plexus, including the cavernous nerves, passes through the rectourethral muscle [29, 30]. Clinically, even after non-nerve sparing prostatectomy, erectile function may be maintained [31, 32], which implies that the cavernous nerves along the posterior side of the prostate and urethra are preserved, in some cases, after resecting the so-called neurovascular bundle. Furthermore, differences in the postoperative recovery of erectile function depend on the quantity of damaged cavernous nerves. Therefore, athermal dissection and reduced traction may lead to the preservation of function, even when non-nerve-sparing procedures are used.

There are several limitations to the present study. Because of the lack of immunohistochemical analysis of the hypogastric and pelvic splanchnic nerves, it was not possible to propose a complete scheme from a preganglionic fiber, via a ganglion cell and postganglionic fibers, to the target organs. Additionally, nNOS immunohistochemistry is difficult to successfully perform in cadaveric specimens that have undergone long periods of preservation [9]. Because the quality of nNOS immunohistochemistry varies among individuals, we did not perform a quantitative evaluation, which might have included a determination of the percentages of each nerve type.

## Conclusions

Because the dominance of VIP-positive nerve fibers varied by site, nerves of the pelvic plexus branches were clearly categorized as those nerves around the bladder and seminal vesicle (VIP-negative) and those nerves around the prostate (VIP-positive). Furthermore, the results confirmed that nNOS expression is a general characteristic of the pelvic plexus branches, rather than a specific characteristic of the cavernous nerve. Although nNOS- and VIP-positive nerve fibers are candidate cavernous nerves, the periprostatic nNOS-positive fibers may not be cavernous nerves, even when they are observed alongside VIP-positive fibers.

## Abbreviations

H&E: hematoxylin and eosin
HRP: horseradish peroxidase
nNOS: nitric oxide synthase
TH: tyrosine hydroxylase
VIP: vasoactive intestinal polypeptide
